# Reconstruction of focal cartilage defects in the talus with miniarthrotomy and collagen matrix

**DOI:** 10.1007/s00064-012-0229-9

**Published:** 2014-06-06

**Authors:** M. Walther, S. Altenberger, S. Kriegelstein, C. Volkering, A. Röser

**Affiliations:** Center for Foot and Ankle Surgery, Schön Klinik München Harlaching, Harlachingerstr. 51, 81547 Munich, Germany

**Keywords:** Osteochondrosis dissecans, Microfracture, Ankle joint, Autograft, Chondrogenesis, Osteochondrosis dissecans, Mikrofraktur, Sprunggelenk, Autologes Transplantat, Chondrogenese

## Abstract

**Surgical principal and objective:**

Treatment of focal cartilage defects (traumatic or osteochondrosis dissecans) of the talus using a collagen matrix. The goal is to stabilize the superclot formed after microfracturing to accommodate cartilage repair. The procedure can be carried out via miniarthrotomy, without medial malleolus osteotomy.

**Indications:**

International Cartilage Repair Society (ICRS) grade III and IV focal cartilage defects of the talus > 1.5 cm^2^.

**Contraindications:**

Generalized osteoarthritis, inflammatory joint disease, gout, neuroarthropathy.

**Surgical technique:**

Miniarthrotomy to open the ankle joint. Debridement of unstable cartilage and necrotic bone, curettage of cysts. Filling of the bone defects with autologous cancellous bone. Sealing of reconstructed bone with fibrin glue and attachment of a collagen matrix shaped to precisely fit the defect.

**Postoperative regimen:**

Immobilization for 48 h. Partial weight bearing of 10 kg for 6 weeks, with continuous passive motion. Increasing weight bearing from 7 weeks onwards.

**Results:**

Follow-up of at least 30 months in 14 patients showed improvement in the Score of the American Orthopedic Foot and Ankle Society (AOFAS) from 50 to 89 points, with equal mobility on both sides of the upper ankle joint.

## Abstract (de)

**Operationsziel:**

Therapie von umschriebenen Knorpeldefekten (traumatisch oder durch Osteochondrosis dissecans) des Talus mit einer Kollagenmatrix. Ziel ist es, den Superclot nach der Mikrofrakturierung zu stabilisieren und die Regeneration von Faserknorpel zu unterstützten. Der Eingriff kann über eine Miniarthrotomie ohne Innenknöchelosteotomie durchgeführt werden.

**Indikationen:**

Umschriebene Knorpeldefekte des Talus (Grad III und IV nach International Cartilage Repair Society, ICRS) > 1,5 cm^2^.

**Kontraindikationen:**

Generalisierte degenerative Gelenkveränderungen, entzündliche Gelenkerkrankung, Gicht, Neuroarthopathie.

**Operationstechnik:**

Eröffnung des Sprunggelenks über eine Miniarthrotomie. Débridement von instabilem Knorpel, nekrotischem Knochen und Zysten. Füllung von Knochendefekten mit autologer Spongiosa. Versiegeln der Spongiosaplastik mit Fibrinkleber und Einkleben einer passgenau zugeschnittenen Kollagenmatrix.

**Weiterbehandlung:**

48 h Ruhigstellung, 6 Wochen Teilbelastung 10 kg und Motorschiene. Ab der 7. Woche stufenweiser Belastungsaufbau.

**Ergebnisse:**

Bei 14 Patienten mit einem Follow-up von mindestens 30 Monaten konnte eine Verbesserung des AOFAS-Scores (American Orthopedic Foot and Ankle Society) von 50 auf 89 Punkte beobachtet werden, bei seitengleicher Beweglichkeit des oberen Sprunggelenks.

## Introduction

Microfracture is the established procedure for treatment of symptomatic International Cartilage Repair Society (ICRS) grade III–IV cartilage defects of the talus [25]. The purpose of subchondral bone perforation, as in microfracture, is to enhance chondral resurfacing by providing a suitable environment for new tissue formation. The released progenitor cells, mesenchymal stem cells, growth factors and other cytokines form a surgically induced superclot, which provides an enriched environment for fibrous cartilage formation. In defects with subchondral cyst formation, microfracture can be combined with cyst resection and filling of the defect with bone graft from the iliac crest, the tibia or the calcaneus. Compared to all other cartilage repair techniques, the level of evidence for good clinical outcome is highest for microfracture [17]. However, the good initial clinical results are reported to begin to lessen after 5 years [11, 19]. Significantly poorer results have been reported for defects exceeding 1.5 cm^2^ [4, 5].

Autologous chondrocyte implantation (ACI) has increasing gained popularity for the treatment of larger defects during the past few years. ACI is a procedure in which cartilage cells harvested from the patient are expanded in vitro and then implanted into the joint in a second intervention. The superiority of this expensive and time-consuming cartilage reconstruction technique over that of microfracture could not be conclusively established so far [9, 10, 20]. Current research is focused on one-step procedures, e.g. scaffold-enhanced microfracture, with the goal of providing a simple, cost-effective clinical solution that avoids the problems associated with cell culture and a second surgical intervention [8, 28].

In larger defects, stabilization of the progenitor cell-containing blood clot formed after microfracture presents a problem [18]. By stabilizing this superclot with a biomaterial, the colonization, proliferation and chondrogenesis of mesenchymal stem cells are facilitated [3, 12].

Autologous matrix-induced chondrogenesis (AMIC) is a cartilage repair technique in which, after microfracturing, a collagen type I/III bilayer matrix (Chondro-Gide®, Geistlich Pharma AG, Switzerland) is secured over the cartilage defect using commercially available fibrin glue. Mesenchymal progenitor cells migrate toward and adhere to the porous layer of the matrix; the cell-occlusive compact layer prevents cell loss through leakage into the joint space and protects cells from mechanical stress. In vitro studies have shown that the stability of the collagen matrix form prevents shrinkage of the super clot. Furthermore, its use in combination with fibrin glue (Tissucol or Tisseel from Baxter Healthcare, Deerfield, IL, USA) supports chondrogenic differentiation of human mesenchymal stem cells and significantly enhances proteoglycan deposition [6, 22].

The controversial in vitro evidence on the effects of fibrin glue on mesenchymal stem cells and chondrocytes has been a topic of discussion for well over a decade. In rabbit experiments, Brittberg et al. [2] found that the use of Tisseel impaired healing of osteocondral defects and also reported that no cell migration into Tisseel took place, whereas cell migration was observed into blood clots. In contrast, Homminga et al. [13] have shown that chondrocytes in fibrin glue produce extracellular matrix, retain their typical morphology and divide to form colonies. Nowadays, commercially available fibrin glue is routinely used in orthopedics. It is also standard in matrix-induced autologous chondrocyte implantation (MACI) and no negative results have been reported. Its use is supported by the findings of Sage et al. [23], who stated that fibrin glue increases proliferation of and glycosaminoglycan production by cells in cartilage chips. Kirilak et al. [15] found that fibrin sealant promotes migration and proliferation of human articular chondrocytes and that thrombin, an active component of fibrin glue, stimulates these processes.

Alternative techniques for treatment of focal cartilage defects in the talus are the osteochondral autograft transfer system (OATS), allograft or implantation of in vitro cultured autologous chondrocytes in ACI [1, 14, 24]. The OATS approach is associated with high donor site morbidity. In more than 50 % of cases, complaints in the knee joint from where the grafts were harvested were reported [26]. Furthermore, an autograft cannot be implanted in the ankle without an osteotomy of the medial or lateral malleolus, which can lead to future complications and late adverse effects. Allograft is hardly available in Europe.

## Surgical principle and objective

The objective of the procedure is treatment of a talar dome osteochondral lesion (OCL) by microfracture in combination with a collagen matrix. The purpose of the collagen matrix is to retain and stabilize the superclot in the defect zone, as well as to protect it from being displaced [3, 12]. In most cases, the collagen matrix can be implanted without having to perform an osteotomy of the medial or lateral malleolus [29].

## Advantages

- Technically simple surgical procedure
- Availability of the collagen matrix as a ready-to-use product with long shelf-life (Chondro-Gide®)
- One-step intervention
- No donor site morbidity
- In almost all cases the matrix can be implanted via miniarthrotomy alone, without the need for osteotomy of the lateral or medial malleolus
- Saves of 90 % of costs compared to cartilage reconstruction with in vitro cultured chondrocytes

## Disadvantages

- Additional costs of the matrix and fibrin glue as compared to microfracture alone

## Indications

- OCL of the talus (ICRS grades III and IV) > 1.5 cm^2^
- Osteochondrosis dissecans
- Instability and axis malalignment do not represent contraindications if these can be addressed along with cartilage reconstruction

## Contraindications

- Generalized degenerative changes in the joint
- Cartilage defects in the corresponding opposite joint surface
- Inflammatory joint disease
- Crystal arthropathy
- Neuroarthopathy
- Inferior results in older patients are more likely, although there is no clear age limit for the procedure

## Patient information

- Lower leg in plaster cast for 2 weeks
- Weeks 1–6: partial weight bearing of 10 kg, continuous passive motion (CPM), physiotherapy
- Weeks 7–12: step-wise increase in weight bearing (about 20 kg every 2 weeks)
- Harvesting cancellous bone (calcaneus, head of tibia, pelvic crest)
- Possible disturbed mobility of ankle with need for surgical revision
- Possible delayed or absent healing of the cancellous bone graft
- Possible delayed or absent fibrocartilage formation
- Possible intolerance to the materials used
- Possible tendency of swelling for several months
- Possible wound healing disturbances, sensibility disturbances and/or circulation disorders that can lead to loss of the foot
- Inability to work: in most cases, working in a sitting position is allowed after 2 weeks and working in a standing position after 12 to 14 weeks. Occupations with greater physical demands can be undertaken within 6 months
- General risks of surgery

## Preoperative workup

- Clinical assessment of joint stability and hindfoot alignment
- X-ray of the ankle in two planes. In instances of clinical suspicion of axial malalignment, additional radiographs of the lower leg with the patient standing on one foot and a radiograph of the hindfoot according to Saltzman are recommended
- MRI of the ankle for assessment of cartilage lesions, possible subchondral cysts, areas of necrotic bone and other accompanying pathologies
- Documentation of circulation in the foot (posterior tibial and dorsalis pedis artery pulse; if needed, Doppler sonography, capillary refill time)
- Documentation of sensibility (distinguish between sharp/dull, two-point discrimination)
- General preparations for surgery. According to guidelines published by the Robert Koch Institute (RKI Guidelines, http://www.rki.de), shaving can be omitted

## Instruments and implants

**Fig. 1:**
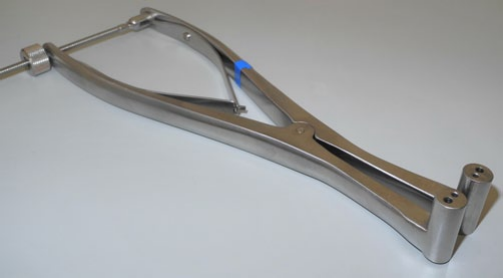
Self-retaining Kirschner wire (K-wire) distractor (courtesy of My medibook GmbH, Berg, Germany)

- Basic set of surgical instruments for ankle surgery
- Self-retaining Kirschner wire (K-wire) distractor (Fig. 1)
- Collagen matrix (Chondro-Gide® in different sizes)
- Fibrin glue (e.g. Tissucol, Baxter Gmbh, Unterschleißheim, Germany)
- Image intensifier if osteotomy or axial correction is planned

 

## Anesthesia and positioning

- Intubation, larynx mask, spinal or conduction anesthesia
- Tourniquet (100 mmHg above the systolic blood pressure) at the upper thigh
- Radiation protection mat from the head down to the middle of the lower thigh
- Supine position

## Surgical technique

Presented in Fig. 2, Fig. 3, Fig. 4, Fig. 5, Fig. 6, Fig. 7, Fig. 8 and Fig. 9.

**Fig. 2:**
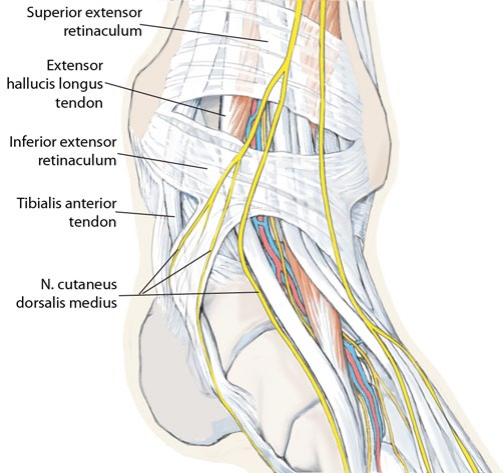
Most osteochondral lesions (OCLs) of the talus are located at the medial talar shoulder. The ventromedial approach is carried out between the medial malleolus and the *anterior tibial tendon (tibialis anterior tendon). N nerve*

**Fig. 3:**
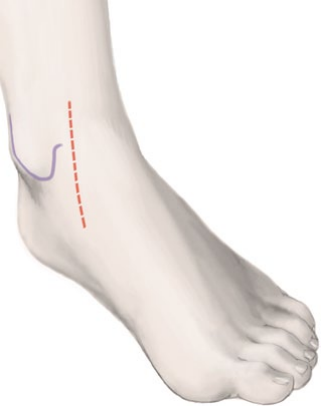
The skin incision is marked medial between the medial malleolus and the anterior tibial tendon, as indicated by the *dashed red line*

**Fig. 4:**
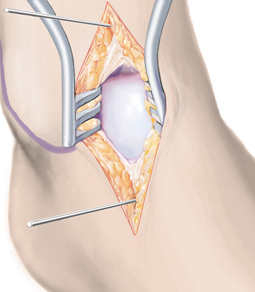
After dissection to the level of the joint capsule, the joint is opened by a longitudinal incision. A 2.0-mm K-wire is drilled into the distal tibia, a second one parallel to the first in the neck of the talus. The talar K-wire is placed at the cartilage-free area ventral to the medial talar shoulder, in order to avoid cartilage damage. Placing the wires using the K-wire distractor as a drill guide facilitates precise positioning of the wires. The joint is then distracted in maximum plantar flexion

**Fig. 5:**
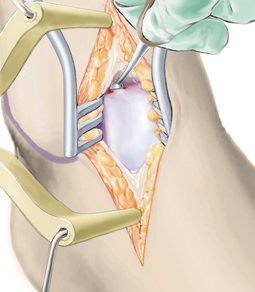
Unstable cartilage is radically debrided. In the border region, a stable cartilage edge must mark the transition to healthy cartilage. Oval-shaped preparation of the defect facilitates insertion of the collagen matrix. All necrotic bone is removed and cysts curetted. The underlying sclerotic zone is then perforated using multiple small drill holes (1.2-mm K-wire) with adequate cooling or microfracture awls. Particularly in cases with a thick sclerotic wall visible in the preoperative MRI, drilling is preferred to completely perforate the sclerosis. In full-thickness cartilage defects without additional sclerosis, microfracturing is sufficient, avoiding thermal damage to the bone

**Fig. 6:**
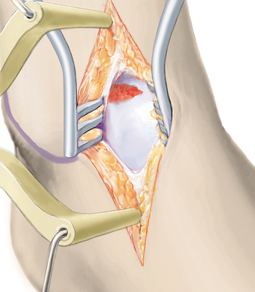
Osseous defects of 2 mm or more are reconstructed to the level of the subchondral bone lamella using autologous cancellous bone. Particular attention must be paid to ensure that the bone graft does not exceed the level of the subchondral bone lamella, which can lead to delamination of the collagen matrix. Cancellous bone harvested from the ipsilateral calcaneus is adequate in most cases. It is also possible to harvest cancellous bone from the tibial head or pelvic crest. Although the content of stem cells in iliac crest bone is higher than in the calcaneus, there is currently no clinical evidence that this has any impact on clinical outcome [7, 21]. The bone graft is sealed with fibrin glue, which provides sufficient stability to reconstruct even the talar shoulder

**Fig. 7:**
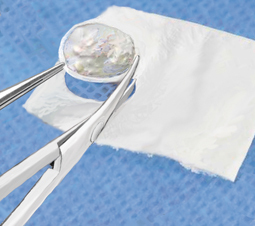
The defect size is measured with the help of an aluminum foil, which is pressed into the defect with forceps such that the borders of the cartilage are clearly depicted. It is then cut to size and its exact fit is checked once again. The collagen matrix, hydrated in a physiological saline solution, is cut to shape with the help of the template. When hydrated, the matrix expands by 10–15 %. The collagen matrix has a rough side that should face the bone; the smooth side faces the joint

**Fig. 8:**
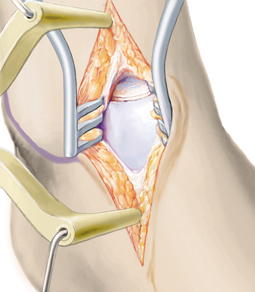
The cancellous bone graft is covered with commercially available fibrin glue and the collagen matrix is glued on top. Additional suturing is not required. To decrease the risk of delamination when the joint is moved, care should be taken not to overlap the matrix with the edges of the adjacent cartilage. After hardening of the glue, the distractor is removed and the joint moved throughout the range of motion several times. If delamination occurs, possible matrix protrusion with the adjacent cartilage should be assessed and eliminated by shaving off excess matrix. Bone graft exceeding the level of the subchondral bone can also lead to delamination of the matrix. In this case, excess graft should be reduced before reattaching the matrix

**Fig. 9:**
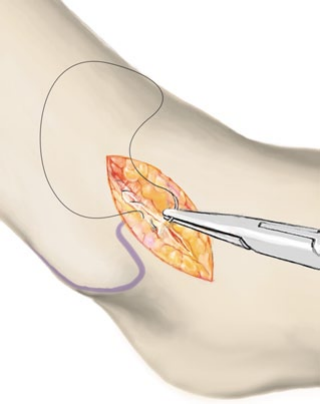
Finally, the joint is closed in layers with resorbable suture material. A drain without suction can be inserted if necessary. After applying an elastic compression bandage, the tourniquet is released. The ankle is immobilized with a dorsal plaster splint for the first 48 h after surgery

## Special surgical considerations

**Fig. 10:**
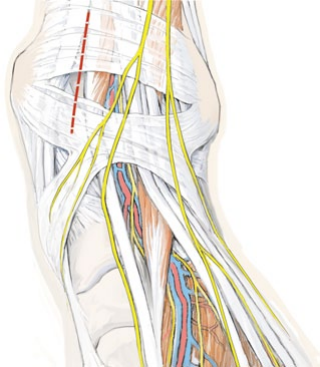
Ventrocentral approach between the anterior tibial and the extensor hallucis longus tendon, as indicated by the *dashed red line*

**Fig. 11:**
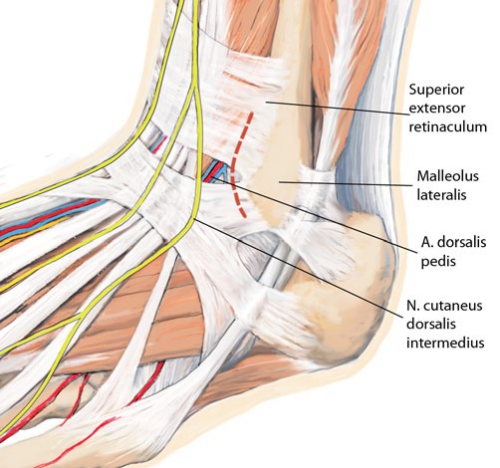
Ventrolateral approach (Ollier approach) lateral to the peroneus terzius tendon and ventral to the lateral malleolus (*malleolus lateralis*), as indicated by the *dashed red line*. *A *artery, *N* nerve

**Fig. 12:**
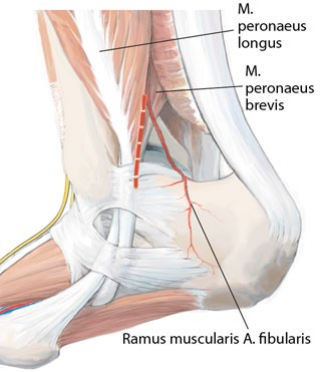
Dorsolateral approach dorsal to the lateral malleolus, as indicated by the *dashed red line*. *M *muscle,* A *artery

**Fig. 13:**
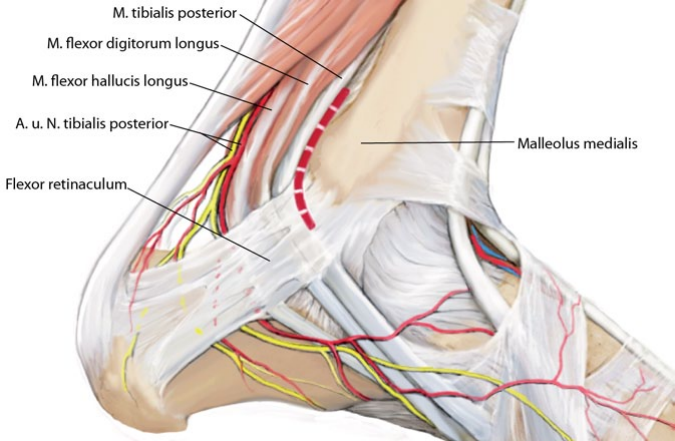
Dorsomedial approach between the medial malleolus (*malleolus medialis*) and the posterior tibial tendon, as indicated by the *dashed red line*. *M *muscle,* A *artery, *N* nerve

**Fig. 14:**
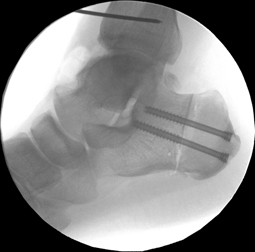
Concurrently performed closing wedge osteotomy of the calcaneus in a patient with hindfoot varus

- Depending on the location of the cartilage defect, other approaches can be used to facilitate access to the diseased cartilage area. With a central approach, the entire ankle joint can be well visualized. This approach is particularly useful for treating defects of the medial and lateral talar shoulder, as well as for centrally located defects. The ventrocentral access approach uses the space between the anterior tibial and the extensor hallucis longus tendons (Fig. 10). The neurovascular bundle is retracted laterally using a blunt Hohmann retractor.
- The ventrolateral approach (Ollier approach) is carried out lateral to the peroneus tertius tendon, ventral to the lateral malleolus (Fig. 11). Lesions of the lateral talar shoulder can be addressed well with this approach. The closure of the capsule can be combined with an external ligaments stabilization procedure.
- Dorsal approaches are seldom necessary, since with adequate distraction of the joint using the K-wire distractor (Fig. 1), more dorsally located defects can also be accessed. If such an approach is planned, the patient is positioned on the contralateral side in the case of a dorsolateral defect. The affected leg is positioned free, in order to be able to flex the knee to allow for adequate dorsal extension of the ankle joint. The dorsolateral approach runs dorsal to the lateral malleolus and enables medial or lateral retraction of the peroneal tendons (Fig. 12).
- For a dorsomedial approach between the medial malleolus and the posterior tibial tendon (Fig. 13), the patient is positioned on the affected side. The contralateral leg is well-padded and the affected leg is positioned in a freely moveable manner. The use of a short vacuum mattress considerably facilitates the procedure. Adequate flexion of the knee is necessary to allow free dorsiflexion of the ankle joint.
- Any axial deformities or instabilities present should be addressed within the context of this intervention. Hindfoot deformity corrections, as well as capsule and ligament reconstructions can be carried out according to standard surgical techniques. Fig. 14 shows a patient with hindfoot varus undergoing a concurrently performed closing wedge osteotomy of the calcaneus.

 

## Postoperative management

- Complete immobilization of the ankle at 90° for 48 h. If inserted, remove drain after 48 h. Begin with continuous passive motion limited to 20–0–20°, to provide continuous containment of the defect by the tibia during the healing period and to minimize the risk of delamination. In defects located very ventral or dorsal to the joint surface, the recommended range of motion is modified accordingly.
- Dorsal splint for 2 weeks until wound healing is complete.
- Weeks 1–6: partial weight bearing of 10 kg.
- Weeks 7–12: stepwise increase in weight bearing (increasing by 20 kg every 2 weeks).
- From week 13 onward, daily activities including cycling and swimming are permitted.
- Sports involving impact load or a rapid change of direction should be avoided for at least 12 months.
- Whether or not a return to professional sports is possible after cartilage reconstruction has not yet been conclusively established.
- There is no indication for routine MRI follow-up. However, if the patient complains of persistent pain, MRI is indicated.

## Errors, hazards and complications

- If the OCL cannot be reached adequately, an additional osteotomy should be considered. Ventral access can be extended by a v-shaped osteotomy of the tibial plafond located directly over the lesion. The dorsomedial approach can be enlarged by a medial malleolar osteotomy; the dorsolateral approach by an osteotomy of the fibula.
- Intraoperative delamination of the matrix can be addressed by elimination of matrix protrusion at the edge of the adjacent cartilage. If the bone graft is too thick, the surface layer should be ablated and the matrix reattached.
- Deep wound infection should be managed by a wound swab, early surgical debridement and appropriate antibiotic treatment. In the case of joint involvement, repeated joint lavage may be necessary until clinical and systemic infection signs regress.
- Incomplete healing of bone graft with recurring cyst formation [27]. Repetition of the intervention is possible. However, after failure of initial surgery, it is important to reassess axial malalignment or instability as possible causes.

## Results

**Fig. 15:**
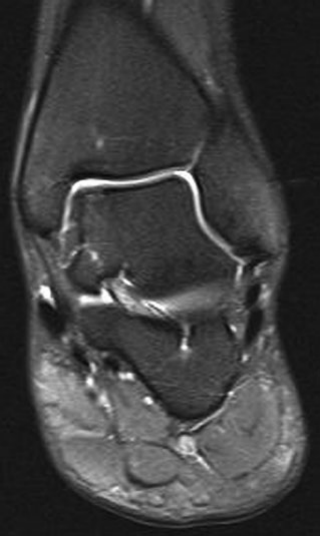
Complete coverage of the former defect at 1-year follow-up evaluation (courtesy of My medibook GmbH)

Postoperative results for a follow-up period of 30 months or more are available for 14 patients with medial talar cartilage defects (ICRS grades III and IV> 1.5 cm^2^). Patients were treated with AMIC as described (Tab. 1). No intraoperative complications were observed. In 10 cases, a cancellous bone graft from the calcaneus was used. The Score of the American Orthopedic Foot and Ankle Society (AOFAS, based on the score established by Kitaoka [16]) improved from 50.1 ± 9.7 to 89.0 ± 9.3. In 2 patients, MRI revealed new cyst formation. In 1 patient, residual complaints necessitated repeat arthroscopy, which showed an unstable graft and hypertrophic repair tissue with impingement. In all other cases, MRI revealed good defect filling without increased effusion (Fig. 15).

**Tab. 1:** Results of 14 patients with cartilage defects of the medial talus treated by a medial approach without osteotomy of the medial malleolus

Initial	Gender	Age (years)	Defect size (cm^2^)	Bone graft	AOFAS pre-OP	Follow-up (months)	AOFAS at follow-up
1	F	24	2.0	Yes	62	37	97
2	F	22	1.5	Yes	47	37	90
3	M	49	1.5	Yes	49	36	85
4	M	40	2.0	Yes	50	36	97
5	F	31	1.5	No	53	35	100
6	M	45	1.5	Yes	71	35	66
7	F	41	1.5	No	53	35	78
8	M	22	2.0	Yes	63	33	87
9	F	29	2.0	Yes	35	33	90
10	M	26	3	No	43	32	87
11	F	49	2.5	No	39	32	97
12	F	57	1.5	Yes	47	31	85
13	F	16	2.0	Yes	47	30	100
14	M	28	3.0	Yes	43	30	87
				Mean	50.14	33.71	89.00
				SD	± 9.78	± 2.46	± 9.32

*F* female, *M* male, *AOFAS* Score of the American Orthopedic Foot and Ankle Society, *pre-OP* preoperative, *SD* standard deviation.

Further sufficiently powered, randomized clinical trials with uniform methodology and validated outcome measures are needed to compare the results of surgical strategies for treating OCLs of the talus. The results suggest that AMIC may be an effective way to treat full-thickness lesions of the talus without harvesting chondrocytes from the talus in patients who do not respond to initial curettage.
